# A Meta-Analysis To Ascertain the Effectiveness of COVID-19 Vaccines on Clinical Outcomes in Patients With COVID-19 Infection in North America

**DOI:** 10.7759/cureus.41053

**Published:** 2023-06-27

**Authors:** Anderson E Ikeokwu, Olanrewaju F Adeniran, Farirai M Marwizi, Oreoluwa J Kolade-Ernest, Rebecca O Solomon, William Ogedengbe, Precious Onyemarin-Henry, Nicodemus C Okpo, Okam Onyinye

**Affiliations:** 1 Medicine, Richmond Gabriel University, Kingstown, VCT; 2 Internal Medicine, West Virginia University, Morgantown, CAN; 3 General Medicine, Universitatea de Medicină şi Farmacie, Timisoara, ROU; 4 Pediatrics, SUNY (State University of New York) Downstate Health Sciences University, New York City, USA; 5 Community Medicine, Lagos State Primary Health Care Board, Lagos, NGA; 6 Medicine and Surgery, Lagos University Teaching Hospital (LUTH), Lagos, NGA; 7 Pediatric Medicine, Asaba Specialist Hospital, Asaba, NGA; 8 Internal Medicine, Shaqra General Hospital, Shaqra, SAU; 9 Internal Medicine, Alimosho General Hospital, Lagos, NGA

**Keywords:** covid-19 vaccine progress, coronavirus disease (covid-19), covid-19 vaccine, vaccination status, incidence rate, icu admissions, mechanical ventilation, rate of hospitalization

## Abstract

The challenges in controlling the pandemic have been exacerbated by the disease’s morbidity and the emergence of additional COVID-19 variants. The use of emergency vaccines to circumvent these challenges has sparked mixed opinions on their effectiveness. Therefore, we conducted a meta-analysis to assess the efficacy of COVID-19 vaccines on clinical outcomes such as incidence, hospitalization, and ventilation rates in both vaccinated and unvaccinated patients. PubMed, Google Scholar, and Cochrane Central Register of Clinical Trials were searched on April 21, 2022, to extract published articles comparing vaccinated COVID-19 patients versus unvaccinated COVID-19 patients and their clinical outcomes. The clinical outcomes studied were incidence rate, intensive care unit (ICU) admission, mechanical ventilation, and hospitalization rates. The analysis was performed with Review Manager (RevMan) software. Random-effect models were used to calculate pooled odds ratio and corresponding 95% confidence interval (CI). In our meta-analysis, we have identified a total of 250 published findings, encompassing 15 studies that involved a cumulative count of 24,164,227 individuals diagnosed with COVID-19. Being unvaccinated had a significant association with severe clinical outcomes in patients infected with COVID-19. Unvaccinated individuals were 2.36 times more likely to be infected, with a 95% CI ranging from 1.13 to 4.94 (p = 0.02). Unvaccinated subjects with COVID-19 infection were 6.93 times more likely to be admitted to the ICU than their vaccinated counterparts, with a 95% CI ranging from 3.57 to 13.46 (p < 0.0001). The hospitalization rate was 3.37 higher among the unvaccinated compared to those vaccinated, with a 95% CI ranging from 1.92 to 5.93 (p < 0.0001). In addition, patients with COVID-19 infection who are unvaccinated were 6.44 times more likely to be mechanically ventilated than those vaccinated, with a 95% CI ranging from 3.13 to 13.23 (p < 0.0001). Overall, our study revealed that vaccination against COVID-19 disease is beneficial and effective in mitigating the spread of the infection and associated clinical outcomes. However, more awareness and proper education must be made to increase vaccine acceptance. We, therefore, recommend and urge all stakeholders involved in COVID-19 prevention, management, and control to strengthen awareness and educate the people on the effectiveness of COVID-19 vaccination.

## Introduction and background

The Coronaviridae family of viruses includes the recently described severe acute respiratory syndrome coronavirus (SARS-CoV-2), which is the underlying etiology of COVID-19. This mRNA virus causes an inflammatory respiratory infection that mainly affects the lower respiratory tract and is marked by a rise in proinflammatory cytokines like interleukin 6 (IL-6) and interferon-gamma (IFN-γ). The symptoms can range from mild to severe acute respiratory distress syndrome, which can also be accompanied by a fulminant systemic inflammatory response, possibly leading to multiorgan failure and death [1].

As new COVID-19 variations emerge, the Centers for Disease Control and Prevention (CDC) reports an ever-changing virus epidemiology. In North America, there have been 91.8 million cases and 1.4 million deaths since the pandemic began, with 82,217,041 cases and 993,959 deaths in the United States [2]. The case fatality ratio in the United States is 1.2%, while it is 1.1% in Canada and 5.6% in Mexico [2]. In North America, a total of 865,103,192 COVID-19 vaccine doses have been administered, with 575,410,180 doses provided in the United States, comprising single and booster doses [2]. According to the US Coronavirus vaccine tracker, 78% of the population has received at least one dose, and 66% received three doses [3]. Thirty-one percent of the population has received at least one booster dose. On the other hand, in Canada and Mexico, 81.4% and 61.2% of their populations received at least two doses, respectively [4].

As a result of the disease's lethality, researchers are constantly working to combat the virus's devastating effects on both the therapeutic and preventive fronts. Vaccination is considered the safest method of preventing future COVID-19 infections and related clinical outcomes. However, the emergency use authorization of COVID-19 vaccines has sparked skepticism and hampered vaccination acceptance. This could be due to a lack of trust in public health policies, concerns about vaccine safety, or a general aversion to vaccination [5]. In addition, the public appears divided about whether vaccination status affects any component of the COVID-19 disease course. Therefore, we conducted a systematic review and meta-analysis to evaluate the relationship between the clinical outcomes and vaccination status in infected subjects to shed more light on this severe infectious disease.

## Review

Materials and methods

Study Design

A meta-analysis of studies was conducted to assess the effectiveness of COVID-19 vaccination. The Preferred Reporting Items for Systematic Reviews and Meta-Analyses (PRISMA) guidelines were followed to review the studied articles [6]. This review was registered in PROSPERO 2022 (359550).

Eligibility Criteria

The inclusion criteria were studies that reported the effectiveness of COVID-19 vaccination on patient outcomes with COVID-19 infection and studies published after the availability of COVID-19 vaccines. All types of COVID-19 vaccines approved in North America were included in this review.

Population (P): We included cross-sectional, case-control, cohort studies, and randomized controlled trials from North America published in English from March 17, 2020, to July 10, 2022. Case series/reports, conference papers, proceedings, articles available only in abstract form, editorial reviews, letters of communication, commentaries, systematic reviews, and qualitative studies were excluded. Articles in languages other than English or study areas beyond North America were also excluded.

Intervention (I): All types of COVID-19 vaccines approved in North America were included in this review.

Comparison (C): We included studies that compared two groups of patients according to their vaccination status. Individuals who received at least one dose of any COVID-19 vaccine were placed in the "vaccinated group," while individuals who did not receive any vaccine were placed in the "unvaccinated group."

Outcomes (O): Our primary outcome measures were incidence, hospitalization, intensive care unit (ICU) admission rates, and the need for mechanical ventilation.

Information Sources

On April 21, 2022, a systematic search was performed. We searched three databases: PubMed, Cochrane, and Google Scholar. A snowball search to identify additional studies was carried out by searching the reference lists of publications eligible for full-text review and using Google Scholar to locate and screen studies citing them. Finally, we updated the database search on July 7, 2022, and the snowball and additional searches on July 8, 2022.

Search Strategy

The search was done using the generic free-text search terms developed based on the Patient/Population, Intervention, Comparable group,/Control, and Outcomes (PICO) model to define the clinical question and aid in finding clinically relevant evidence in the literature. P = "COVID-19" AND "NORTH AMERICA, I = "COVID-19 VACCINE, C = "VACCINATION STATUS" OR "VACCINATED" AND "UNVACCINATED," O = "INCIDENCE RATE" OR "NUMBER OF DAYS ILL" OR "NO OF DAYS ON ADMISSION, "OR "NEED FOR ASSISTED VENTILATION" OR "MORTALITY." The search terms were kept broad to encompass all possibilities for applicable studies. All studies published from March 17, 2020, to July 10, 2022, were retrieved to assess their eligibility for inclusion in this study. We limited our search to full-text articles in the English language. To find additional eligible studies, reference lists of included citations were cross-checked.

Selection Process

All records identified by our search strategy were exported to Rayyan Intelligent Systematic Review software (Rayyan System Inc. Cambridge, Massachusetts, USA). Duplicate articles were removed from the list. Three researchers (AO, AI, and OP) independently reviewed the title and abstract of the first 100 records and discussed inconsistencies until consensus was obtained. Then, in pairs, the researchers screened the titles and abstracts of all articles retrieved. In the case of disagreement, a final agreement on which articles to screen full-text was made by discussion. When necessary, the third researcher was consulted to make the final decision.

**Figure 1:**
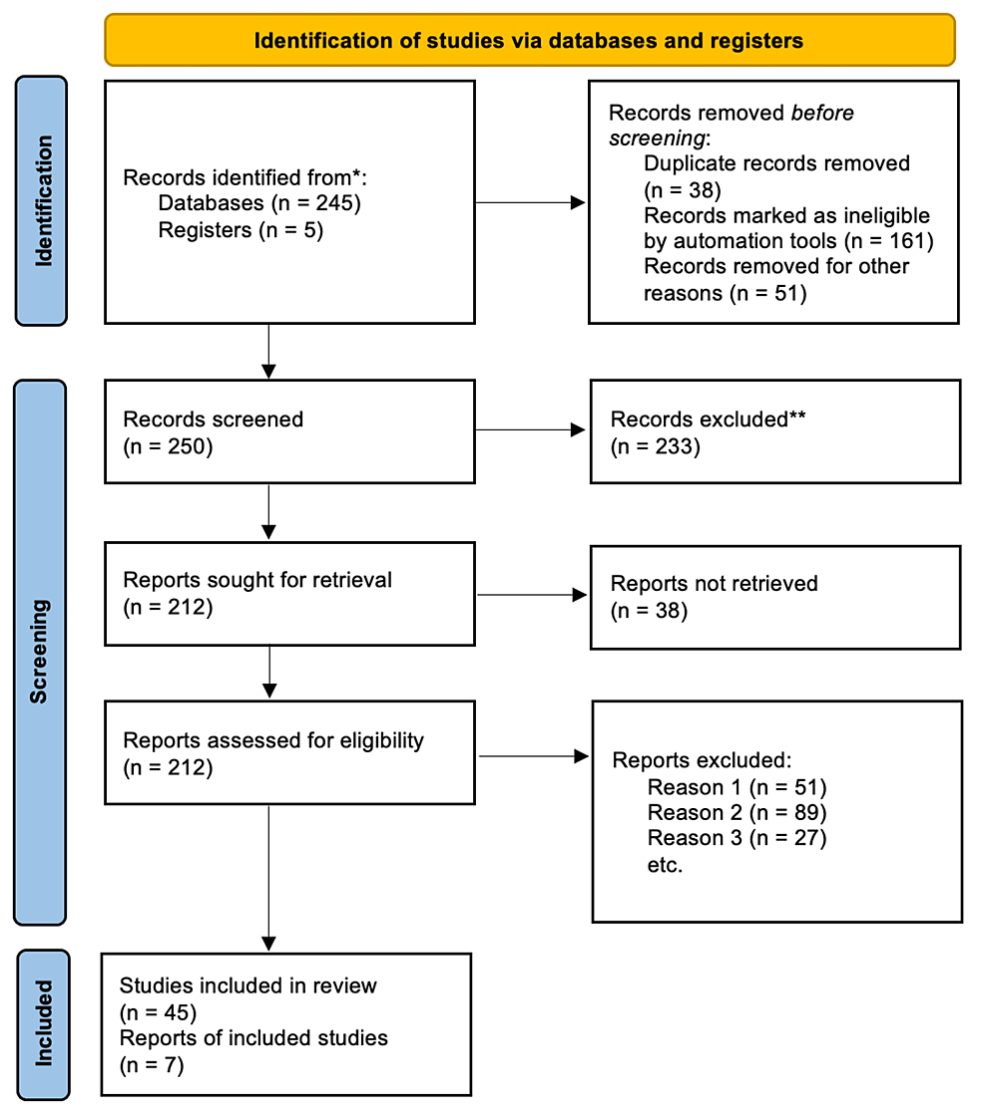
Flow diagram of the inclusion criteria of studies found eligible in the meta-analysis * indicates studies reviewed. ** indicates studies excluded.

Additionally, two researchers (referred to as AO and AI) independently evaluated complete articles to determine their suitability for inclusion. In instances where there were disagreements, a consensus on eligibility was reached through discussion, and if necessary, a third researcher (OP) was consulted. The search methodology was documented in the PRISMA flow chart, which depicted the studies that were included as well as those excluded with accompanying justifications. Exclusion reasons included: reason 1: absence of comparable groups (i.e., vaccinated vs. unvaccinated), reason 2: unavailability of the complete text, and reason 3: lack of relevance to the research question, encompassing insufficient data on patient health outcomes, as illustrated in Figure 1.

Data Collection Process

We designed a data extraction form that two review authors (MFM and KO) used to extract data from eligible studies. Extracted data were compared, with discrepancies resolved through discussion. KO entered data into Review Manager 5 software (Review Manager 2014), double-checking this for accuracy. 

Data Outcomes

The data included the first author, year of publication, study location, study design, setting, population characteristics of the COVID-19 patients with various comorbidities, number of doses, sample size, proportion, and data to calculate effect estimates. The outcomes recorded for the meta-analysis were the incidence, hospitalization, ICU admission, and need for assisted ventilation due to COVID-19 infection. The incidence was defined as the number of new COVID-19 infections, considering the population at risk since 2020 [7]. Hospitalization with COVID-19 was defined as admission within 14 days of testing positive for SARS-CoV-2 via PCR test and included those who tested positive within two days of discharge [7]. Assisted ventilation was defined as using devices to support respiration in hospitalized COVID-19 patients [7]. Finally, ICU admission was defined as the number of patients admitted into the ICU after a positive PCR test for COVID-19.

Effect Measures

The effectiveness of COVID-19 vaccination on infected patients was reported in pooled estimate proportion with a 95% confidence interval. In addition, we analyzed dichotomous outcomes by calculating each study's risk ratio (RR) of a patient outcome (i.e., incidence, hospitalization, ICU admission, and need for mechanical ventilation).

Synthesis Methods

The analysis was performed with Review Manager (RevMan, RevMan International Inc., New York, USA) software. First, a generic inverse variance with a random-effects model was applied to pool the proportion of the studies' data. The heterogeneity was assessed by I^2^ statistic and p-value. If the p-value is < 0.05 or I^2^ > 50%, the assumption of homogeneity was rejected, and a random-effects model was adopted. 

Study Risk of Bias Assessment 

Critical appraisal for data quality was assessed using the Joanna Briggs Institute (J.B.I.) meta-analysis for cross-sectional, case-control, cohort studies, and randomized clinical trials. The risk of bias for the observation study (case-control and cohort) was assessed by nine criteria: appropriateness of the sample frame; appropriateness of study participants sampled; adequate sample size, description of study subjects and the setting; sample size justification; power description, or variance and effect estimates; valid methods for the identification of the condition; a standard and reliable requirement measured; appropriateness of statistical analysis; and adequate response rate. The criteria of the risk assessment were represented by "yes," "no," "unclear," or "not available." The score for yes was one (1) and zero (0) for the rest. The risk of bias was considered low when the total score was more than 70%, moderate when 50%-69%, and high when up to 0-49% (ref). Two authors performed bias assessments independently.

Results

We identified 250 published papers in database searching. These articles included a total of 24,164,227 COVID-19 patients. From the initial search, 240 articles in total were identified from PubMed and 10 from the Cochrane database. After duplicate removal, 167 articles were excluded based on the inclusion and exclusion criteria. We finally selected 14 articles for the meta-analysis (Table 1).

**Table 1:** Sample size of selected studies and their characteristics MMWR: *Morbidity and Mortality Weekly Report*, CDC: Centers for Disease Control and Prevention, MR: mortality rate, ICU: intensive care unit, HR: hospitalization, MV: need for mechanical ventilation, CC: COVID cases, VT: tidal volume.

Author (Year)	Study Area	Study Type Design	Type of Journal	Total Number of Patients	Outcomes Analyzed	Vaccine Type
Corchado-Garcia et al. (2021) [8]	USA	Retrospective cohort	JAMA Network	97,787	CC	Janssen (J&J)
Naleway et al. (2021) [9]	USA	Retrospective cohort	*MMWR* (CDC)	482,464	HR, MV, CC, ICU, MR	Pfizer, Moderna, Janssen (J&J)
Johnson et al. (2022) [10]	USA	Retrospective cohort	*MMWR* (CDC)	9,678,557	CC, MR	Unspecified
Danza et al. (2022) [11]	USA	Cross-sectional	*MMWR* (CDC)	422,966	HR, MV, ICU, MR,	Pfizer, Moderna, Janssen (J&J)
White et al. (2021) [12]	USA	Retrospective cohort	The New England Journal of Medicine	22,232	CC	Pfizer, Moderna
Dunkle et al. (2022) [13]	USA, Mexico	Randomized control trial	The New England Journal of Medicine	25,452	CC	Novavax
Olson et al. (2022) [14]	USA	Case-control	The New England Journal of Medicine	1,222	HR, VT, ICU, MR	Pfizer
Polinski et al. (2022) [15]	USA	Retrospective cohort	JAMA Network	2,067,431	HR, CC	Janssen (J&J)
Griffin et al. (2021) [16]	USA	Cross-sectional	*MMWR* (CDC)	43,127	HR, MV, CC, ICU, MR	Pfizer, Moderna, Janssen (J&J)
Tenforde et al. (2021) [17]	USA	Case-control	*MMWR* (CDC)	1,440		Unspecified
Taylor et al. (2021) [18]	USA	Cross-sectional	*MMWR* (CDC)	7,615		Unspecified
Chung et al. (2021) [19]	Canada	Cross-sectional	The BMJ	324,033	CC	Unspecified
Tenforde et al. (2022) [20]	USA	Case-control	JAMA Network	1,983	HR, MR	Pfizer, Moderna
Xu et al. (2022) [21]	USA	Retrospective cohort	*MMWR* (CDC)	10,987,919	MR	Pfizer, Moderna, Janssen (J&J)

In a review examining the effectiveness of COVID-19 vaccination on patient outcomes, the authors designed tables presenting each included study, the citations, study design, country, sample size, median age, gender, and ethnic distribution of vaccinated and unvaccinated patients (Tables 2-4). Patient comorbidities and types of COVID-19 vaccine used for various studies have also been elaborated in Tables 5, 6. In this analysis, the incidence of COVID-19 infection, hospitalization, ICU admission, and mechanical ventilation in various studies are considered clinical outcomes in patients with COVID-19 infection.

**Table 2:** Summary of data on demographics

Author (Year)	Total Number of Patients	N (%) Vaccinated	N (%) Unvaccinated	N (%) Female	N (%) Male	Age Range of Patients
Corchado-Garcia et al. (2021) [8]	97,787	8,889 (9.1)	88,898 (90.9)	48,537 (49.6)	49,239 (50.4)	18-75
Naleway et al. (2021) [9]	482,464	344,848 (71.5)	137,616 (28.5)	251,552 (52.1)	230,552 (47.8)	18-75
Johnson et al. (2022) [10]	9,678,557	2,866,517 (29.6)	6,812,040 (70.4)	-	-	18-65+
Danza et al. (2022) [11]	422,966	281,038 (66.4)	141,928 (33.6)	224,173 (53)	184,134 (43.5)	18-80+
White et al. (2021) [12]	22,232	18,242 (82.1)	3,990 (17.9)	-	-	
Dunkle et al. (2022) [13]	25,452	17,312 (68)	8,140 (32)	12,271 (48.2)	13,181 (51.8)	18-95
Olson et al. (2022) [14]	1,222	345 (28.2)	868 (71.8)	-	-	12-18
Polinski et al. (2022) [15]	2,067,431	422,034 (20.4)	1,645,397 (79.6)	1,159,374 (56)	908,057 (43.9)	18+
Griffin et al. (2021) [16]	43,127	12,326 (28.6)	30,801 (71.4)	21,743 (50.4)	20,425 (47.4)	16-80+
Tenforde et al. (2021) [17]	1,440	307 (21.3)	1,133 (78.7)	598 (41.5)	842 (58.5)	18+
Taylor et al. (2021) [18]	7,615	782 (10.3)	6,061 (89.7)	3,255 (42.7)	3,568 (46.9)	18-65+
Chung et al. (2021) [19]	324,033	21,272 (6.6)	302,761 (93.4)	185,539 (57.3)	138,494 (42.7)	16-80+
Tenforde et al. (2022) [20]	1,983	314 (15.8)	1,669 (84.2)	969 (48.9)	1,014 (51.1)	18-65+
Xu et al. (2022) [21]	10,987,919	6,398,361 (58.2)	4,589,557 (41.8)	5,946,533 (54.1)	5,041,385 (45.9)	12-85+

**Table 3:** Summary of data on demographics of vaccinated patients

Author (Year)	N (%) Vaccinated	N (%) Female	N (%) Male	Mean/Median Age	N (%) White	N (%) Asian	N (%) Black	N (%) Hispanic	N (%) Native American	N (%) Native Hawaiian/Pacific Islander	N (%) Multiple Races/Others/Unknown
Corchado-Garcia et al. (2021) [8]	8,889	4,397 (49.5)	4,491 (50.5)	52.4±16.9	7,945 (89.4)	191 (2.1)	274 (3.1)	-	31 (0.3)	12 (0.1)	436 (4.9)
Naleway et al. (2021) [9]	344,848	187,711 (54.5)	156,960 (45.5)	50	242,110 (70.2)	22,828 (6.6)	8,224 (2.4)	-	12,880 (0.4)	1,931 (0.6)	68,475 (19.9)
Johnson et al. (2022) [10]	2,866,517	-		-	-	-	-	-	-	-	-
Danza et al. (2022) [11]	281,038	154,791 (55.1)	117,971 (42)	36	46,612 (16.6)	26 384 (9.4)	15,991 (5.7)	-	530 (0.2)	2,348 (0.8)	40,538 (14.4)
White et al. (2021) [12]	18,242	-	-	-	-	-	-	-	-	-	-
Dunkle et al. (2022) [13]	17,312	8,262 (47.7)	9,050 (52.3)	47	13,140 (75.9)	761 (4.4)	1,893 (10.9)	-	1,074 (6.2)	47 (0.3)	397 (2.3)
Olson et al. (2022) [14]	345	-	-	16	143 (41.4)	-	68 (19.7)	94 (27.2)	-	-	49 (14.2)
Polinski et al. (2022) [15]	422,034	236,437 (56)	185,597 (44)	54.7±17.4	-	-	-	-	-	-	-
Griffin et al. (2021) [16]	12,326	6,271 (50.9)	5,908 (47.9)	36	3,718 (30.2)	1,009 (8.2)	819 (6.6)	3 961 (32.1)	19 (0.2)	49 (0.4)	2,447 (19.9)
Tenforde et al. (2021) [17]	307	135 (44)	172 (56)	69	191 (62.2)	-	49 (16)	47 (15.4)	-	-	20 (6.5)
Taylor et al. (2021) [18]	782	358 (45.8)	424 (54.2)	-	480 (61.4)	48 (6.1)	157 (20.1)	85 (10.9)	11 (1.4)	-	-
Chung et al. (2021) [19]	21,272	15,259 (71.7)	6,013 (28.3)	51.8	-	-	-	-	-	-	-
Tenforde et al. (2022) [20]	314	138 (44)	176 (56)	67	201 (64)	-	55 (17.5)	44 (14)	-	-	14 (4.5)
Xu et al. (2022) [21]	6,398,361	3,448,362 (53.9)	2,949,999 (46.1)	-	2,778,730 (43.4)	633,212 (10)	341,189 (5.3)	1.409,187 (22)	-	-	880,523 (13.8)

**Table 4:** Summary of data on demographics of unvaccinated patients

Author (Year)	N (%) Unvaccinated	N (%) Female	N (%) Male	Mean/Median Age	N (%) White	N (%) Asian	N (%) Black	N (%) Hispanic	N (%) Native American	N (%) Native Hawaiian/Pacific Islander	N (%) Multiple Races/Others/Unknown
Corchado-Garcia et al. (2021) [8]	88,898	44,140 (49.7)	44,748 (50.3)	51.7±16.7	79,692 (89.6)	1,605 (1.8)	2 281 (2.6)	-	267 (0.3)	100 (0.1)	3,953 (4.4)
Naleway et al. (2021) [9]	137,616	63,841 (46.4)	73,592 (53.5)	37	83,474 (60.7)	3,930 (2.9)	4 851 (3.5)	-	588 (0.4)	1,021 (0.7)	43,752 (31.8)
Johnson et al. (2022) [10]	6,812,040	-	-	-	-	-	-	-	-	-	-
Danza et al. (2022) [11]	141,928	69,382 (48.9)	66,163 (46.6)	35	20,529 (14.5)	7,451 (5.2)	12,319 (8.7)	-	342 (0.2)	1,429 (1)	19,214 (1305)
White et al. (2021) [12]	3,990	-	-	-	-	-	-	-	-	-	-
Dunkle et al. (2022) [13]	8,140	4,009 (49.3)	4,131 (50.7)	47	6,184 (76)	366 (4.5)	900 (11)	-	498 (6.1)	10 (0.1)	177 (2.2)
Olson et al. (2022) [14]	868	-	-	15	358 (41.2)	-	197 (22.7)	191 (22)	-	-	122(14)
Polinski et al. (2022) [15]	1,645,397	922,937 (56.1)	722,460 (43.9)	54.5±17.5	-	-	-	-	-	-	-
Griffin et al. (2021) [16]	30,801	15,472 (50.2)	14,517 (47.1)	32	5,620 (18.2)	961 (3.1)	4,755 (15.4)	10,183 (33.1)	51 (0.2)	161 (0.5)	8,551 (27.8)
Tenforde et al. (2021) [17]	1,133	463 (40.9)	670 (59.1)	55	638 (56.3)	-	200 (17.7)	200 (17.7)	-	-	95 (8.4)
Taylor et al. (2021) [18]	6,061	2,897 (47.8)	3,144 (51.9)	-	2,894 (47.7)	405 (6.7)	1 839 (30.3)	841 (13.9)	63 (1)	-	-
Chung et al. (2021) [19]	302,761	170,280 (56.2)	132,481 (43.8)		-	-	-	-	-	-	-
Tenforde et al. (2022) [20]	1,669	831 (49.8)	838 (50.2)	53	717 (43)	-	453 (27.1)	381 (22.8)	-	-	118 (7.1)
Xu et al. (2022) [21]	4,589,557	2,498,171 (54.4)	2,091,386 (45.6)	-	1,982,834 (43.2)	633,212 (13.8)	262,766 (5.7)	1,201,784 (26.2)	-	-	508,961 (11.1)

**Table 5:** Summary of patient comorbidities for vaccinated patients

Author (Year)	Total number vaccinated	N (%) chronic kidney disease	N (%) Diabetes	N (%) Chronic lung disease	N (%) cardiovascular disease	N (%) Immunodeficiency disorder	N (%) Neuromuscular/Neurological disorder
Corchado-Garcia et al. (2021) [8]	8,889	-	-	-	-	-	-
Naleway et al. (2021) [9]	344,848	32 (0.009)	24 (0.007)	24 (0.007)	-	-	-
Johnson et al. (2022) [10]	2,866,517	-	-	-	-	-	10 (0.0003)
Danza et al. (2022) [11]	281,038	-	-	-	-	-	-
White et al. (2021) [12]	18,242	-	-	-	-	-	-
Dunkle et al. (2022) [13]	17,312	-	-	-	-	-	-
Olson et al. (2022) [14]	345	-	28 (8.1)	81 (23.5)	27 (7.8)	-	-
Polinski et al. (2022) [15]	422,034	21,904 (5.2)	69,272 (16.5)	50,486 (12)	191,134 (45.3)	1,563 (0.4)	121,537 (28.8)
Griffin et al. (2021) [16]	12,326	-	-	-	-	-	-
Tenforde et al. (2021) [17]	307	-	140 (45.6)	91 (29.6)	252 (82)	123 (40)	-
Taylor et al. (2021) [18]	782	-	-	-	-	-	-
Chung et al. (2021) [19]	21,272	-	-	-	-	-	-
Tenforde et al. (2022) [20]	314	-	112 (35.7)	100 (31.8)	236 (75.2)	128 (40.8)	-
Xu et al. (2022) [21]	6,398,361	-	-	-	-	-	-

**Table 6:** Summary of patient comorbidities for unvaccinated patients

Author (Year)	N (%) Unvaccinated	N (%) Chronic Kidney Disease	N (%) Diabetes	N (%) Chronic Lung Disease	N (%) Cardiovascular Disease	N (%) Immunodeficiency Disorder	N (%) Neuromuscular/Neurological Disorder
Corchado-Garcia et al (2021) [8]	88,898	-	-	-	-	-	-
Naleway et al (2021) [9]	137,616	37 (0.03)	98 (0.07)	22 (0.02)	-	-	-
Johnson et al. (2022) [10]	6,812,040	-	-	-	-	-	15 (0.0002)
Danza et al. (2022) [11]	141,928	-	-	-	-	-	-
White et al. (2021) [12]	3,990	-	-	-	-	-	-
Dunkle et al. (2022) [13]	8,140	-	-	-	-	-	-
Olson et al. (2022) [14]	868	-	72 (8.3)	241 (27.8)	69 (8)	-	-
Polinski et al. (2022) [15]	1,645,397	83,640 (5.08)	267,659 (16.3)	197,398 (12)	741,703 (45.1)	6,255 (0.38)	472,062 (28.7)
Griffin et al. (2021) [16]	30,801	-	-	-	-	-	-
Tenforde et al. (2021) [17]	1,133	-	323 (28.5)	213 (18.8)	571 (50.4)	109 (9.6)	-
Taylor et al. (2021) [18]	6,061	-	-	-	-	-	-
Chung et al. (2021) [19]	302,761	-	-	-	-	-	-
Tenforde et al. (2022) [20]	1,669	-	425 (25.5)	327 (19.6)	814 (48.8)	191 (11.4)	-
Xu et al. (2022) [21]	4,589,557	-	-	-	-	-	-

**Figure 2:**
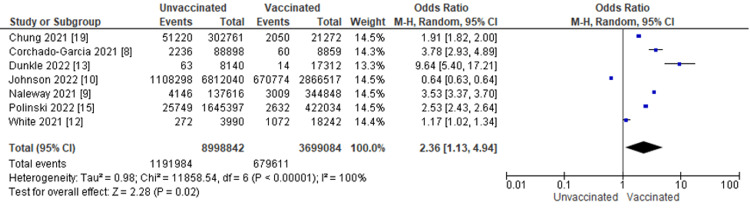
COVID-19 infection by vaccination status M-H: Mantel-Haenszel statistics.

Figure 2 displays for each study included in the meta-analysis the summary statistics (number of events and sample size) for the unvaccinated and vaccinated groups, the odds ratio and its 95% confidence interval, heterogeneity, and test for overall effect for the dichotomous outcome, and the incidence of COVID-19 infection. COVID-19 patients were compared between those who received the COVID-19 vaccine and those who did not. Seven studies, including 1,871,595 patients, reported having COVID-19 infection. The odds ratio of COVID-19 infection between patients with COVID-19 vaccination and patients without was 2.36, with a 95% CI ranging from 1.13 to 4.94. The result was statistically significant, indicating that unvaccinated patients with COVID-19 infection are 2.36 times more likely to have COVID-19 infection if exposed to the virus than vaccinated patients (p = 0.02). In addition, we did a heterogeneity test with results of I^2 ^= 100%, p ≤ 0.00001.

**Figure 3:**
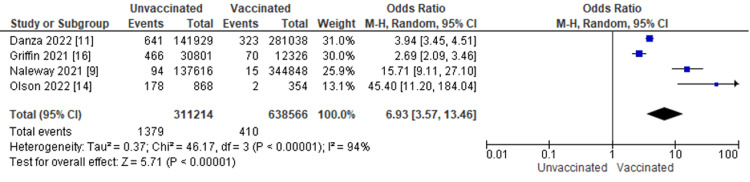
Intensive care unit (ICU) admission from COVID-19 infection by vaccination status M-H: Mantel-Haenszel statistics.

Figure 3 displays for each study included in the meta-analysis the summary statistics (number of events and sample size) for the unvaccinated and vaccinated groups, the odds ratio and its 95% confidence interval, heterogeneity, and test for overall effect for the dichotomous outcome, and ICU admission from COVID-19 infection. Clinical outcomes in COVID-19 patients were compared between the vaccinated and unvaccinated subjects. Four studies, including 1,789 patients, reported being admitted to the ICU. The odds ratio of ICU admission between patients with COVID-19 vaccination and patients without COVID-19 vaccination was 6.93, with a 95% CI ranging from 3.57 to 13.46. The result was statistically significant, indicating that unvaccinated patients are six times more likely to be admitted to the ICU than their vaccinated counterparts (p < 0.0001). In addition, we did a heterogeneity test with results of I^2 ^= 94%, p ≤ 0.00001.

**Figure 4:**
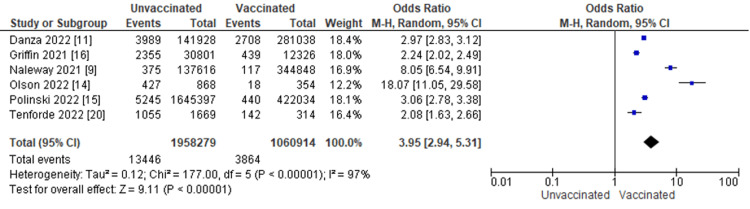
Hospitalization from COVID-19 infection by vaccination status M-H: Mantel-Haenszel statistics.

Figure 4 displays for each study included in the meta-analysis the summary statistics (number of events and sample size) for the unvaccinated and vaccinated groups, the odds ratio and its 95% confidence interval, heterogeneity, and test for overall effect for the dichotomous outcome, and hospitalization from COVID-19 infection. Patient outcomes of COVID-19 infection were compared between vaccinated and unvaccinated individuals. Seven studies, including 55,258 patients, reported hospitalization for COVID-19 infection. The odds ratio of hospitalization from COVID-19 infection between patients with COVID-19 vaccination and those without was 3.37, with a 95% CI ranging from 1.92 to 5.93. The result was statistically significant, which indicates that patients with COVID-19 infection who are unvaccinated are 3.37 times more likely to be hospitalized from COVID-19 infection compared to those with COVID-19 infection who are vaccinated (p < 0.0001). In addition, we did a heterogeneity test with results of I^2 ^= 100%, p ≤ 0.00001.

**Figure 5:**
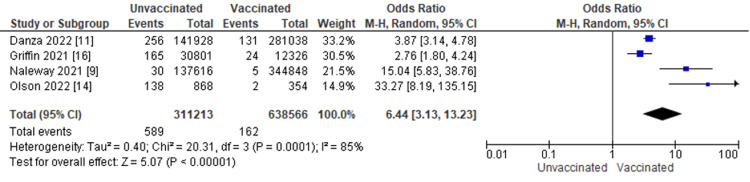
Need for mechanical ventilation from COVID-19 infection by vaccination status M-H: Mantel-Haenszel statistics.

Figure 5 displays for each study included in the meta-analysis the summary statistics (number of events and sample size) for the unvaccinated and vaccinated groups, the odds ratio and its 95% confidence interval, heterogeneity, and test for overall effect for the dichotomous outcome, and need for mechanical ventilation from COVID-19 infection. Clinical outcomes in COVID-19 patients were compared between vaccinated and unvaccinated individuals. Four studies, including 751 patients, reported the need for mechanical ventilation. The odds ratio of mechanical ventilation between patients with COVID-19 vaccination and patients without COVID-19 vaccination was 6.44, with a 95% CI ranging from 3.13 to 13.23. The result was statistically significant, which indicates that patients with COVID-19 infection who are unvaccinated are 6.44 times more likely to be mechanically ventilated than those with COVID-19 infection who are vaccinated (p < 0.0001). In addition, we did a heterogeneity test with results of I^2 ^= 85%, p = 0.0001.

Discussion

This study evaluated the effectiveness of COVID-19 vaccination on patients with COVID-19 infection in North America. In this evaluation, several outcomes were analyzed among individuals aged 12-95 years using the following study designs: retrospective cohort, cross-sectional, case-control studies, and randomized control trials. These studies reveal that COVID-19 vaccination confers a certain level of protection against poor outcomes in COVID-19-infected individuals.

Incidence (Infection Rates)

Among patients infected with the COVID-19 disease, being unvaccinated had a higher likelihood of having poorer health outcomes. In addition, unvaccinated individuals had a higher risk of contracting COVID-19 infection, suggesting that vaccines play an important role in boosting the body's immune system and decreasing the severity of the infection in affected patients [8,9].

Comparing the incidence rate of COVID-19 infection among vaccinated and unvaccinated patients in North America, our analysis demonstrated a strong association between unvaccinated patients and COVID-19 infection. Specifically, our study showed that unvaccinated patients are 2.36 times more likely to have COVID-19 infection if exposed to the virus than vaccinated patients. This finding is consistent with the results of studies that highlighted that the risk of COVID-19 infection was highest for unvaccinated persons, and COVID-19 vaccines were protective against COVID-19 infection [10-13]. Also, a similar study demonstrated that the incidence rate among fully vaccinated persons was three times lower than in unvaccinated persons across all sex, race, ethnicity, and age groups evaluated in the study. In addition, another similar retrospective cohort study showed that a single dose of the COVID-19 vaccine effectively protected against recorded COVID-19 infections [14,15]. However, COVID-19 infections were observed in vaccinated individuals but were rare (0.7% of vaccinated individuals) [8]. Most studies that analyzed the incidence rate of COVID-19 infection suggested similar outcomes; however, a retrospective cohort study showed a substantial case rate increase in COVID-19 infection recorded among both vaccinated and unvaccinated persons [10,15]. However, the findings in this study are subject to at least two limitations; first, variable data linkage completeness might have resulted in misclassification, and second, their data represented 62% of the overall US population and, therefore, might not be generalizable.

Hospitalization Rates

COVID-19 vaccination plays a significant role in reducing hospital admission. Numerous other literature reviews and meta-analytical studies across the globe echo the same outcomes. All six studies that analyzed hospitalization rates by vaccination status consistently showed statistically significantly higher odds of hospitalization in the unvaccinated than the vaccinated, individually and collectively [14-20]. In addition, the mean age of individuals hospitalized with COVID-19 infection was generally higher among vaccinated people than among unvaccinated. While this systematic review did not stratify the efficacy of the COVID-19 vaccine on hospitalization rate according to the number of vaccine doses received or vaccine type, the literature indicates that mRNA vaccines have an efficacy rate of 85% on hospitalizations with COVID-19 [17]. COVID-19 vaccine has recounted a variability of protection after a single dose of vaccine with an average duration of about 180 days, accounting for the reduced hospitalization in participants with at least a single dose [22]. Another study revealed significantly lower hospitalization rates after at least two doses of the COVID-19 vaccine [22].

Further evidence shows that booster shots provide even better protection against hospitalization from COVID-19 infection as they prolong the duration of protection [11]. Booster shots also offer added protection against certain COVID-19 variants [11]. The hospitalization rate was also generally higher among seniors (65+ years) and immunocompromised individuals due to a higher prevalence of comorbidities in the respective subgroups. Comorbidities were associated with a higher hospitalization rate, even in younger subjects. However, COVID-19 vaccination significantly reduced hospital admission rates and conferred better outcomes for this subpopulation [11,17].

ICU Admissions

After examining the effectiveness of vaccination on the rate of ICU admission for COVID-19-infected patients, our analysis showed that patients who had at least one dose of the vaccine types approved in North America are less likely to develop symptoms that warrant ICU admission and possible supportive care. Specifically, unvaccinated patients are six times more likely to require ICU admission than vaccinated patients across the age group of 18-80. These outcomes were less common in fully vaccinated persons with a booster (0.08%, and 0.03%, respectively) and fully vaccinated persons without a booster (0.12% and 0.05%, respectively) [11]. The findings strengthen and augment evidence from previous meta-analyses, which confirmed that single and double doses of COVID-19 vaccines prevented ICU admission rates at 45% and 96%, respectively [20]. Although the World Health Organization (WHO) and other health bodies have advised at least two doses of the vaccine to offer protection to new and emerging variants, this was not very practical as several countries had shortages of vaccine supply at the peak of the pandemic and could only offer the second dose several weeks after the first dose, for example, Canada had to wait 16 weeks after the first doses were administered.

However, recent literature has shown that the T cell and antibody responses induced by a single dose of the SARS-CoV-2 vaccine were comparable to those occurring in subjects naturally infected with SARS-CoV-2 within weeks or months after infection, re-iterating the protection acquired from even single doses of the vaccine on ICU admission [20]. However, multiple doses of the vaccine have been shown to offer protection against ICU admission and mechanical ventilation, as described by a study that showed that unvaccinated persons were more likely to be admitted to an ICU (0.5%) and require intubation for mechanical ventilation (0.2%).

Mechanical Ventilation

In this study, unvaccinated patients had a higher risk of getting intubated for mechanical ventilation than vaccinated patients, confirming several research studies that reported not getting vaccinated as a risk factor for poor outcomes in COVID-19-infected patients [23]. One possible explanation is that the vaccine produces antibodies that tag the virus and neutralize it, preventing the respiratory system from being damaged. These findings align with recent studies [11], highlighting the importance of the COVID-19 vaccine in protecting against severe COVID-19 infection and alleviating the symptoms that could occur. Efforts channeled into promoting the COVID-19 vaccine and boosters, in coordination with other prevention strategies, would go a long way in preventing the need for mechanical ventilation.

Limitations

The risks of COVID-19 infection are not equal for everyone, as the likelihood of exposure might influence the likelihood of COVID-19 vaccine acceptance and coverage. These discrepancies were not accounted for in our study. Independent analyses of the preventive effect of single doses compared to double and booster doses were not performed. Also, the protective development of the vaccines accepted in North America against different virus variants could not be ascertained. Possible reasons could have been the lack of uniformity in vaccine scheduling and availability in North America.

## Conclusions

There has been a lot of skepticism about the efficacy of the COVID-19 vaccines in the population due to the speed of the vaccine rollout. This has affected vaccination acceptance, and this study was done to add to the literature on the effectiveness of COVID-19 vaccination, thereby hoping to increase confidence in acceptance. This meta-analysis showed that COVID-19 vaccination is highly effective in reducing clinical outcomes such as incidence of infection, hospitalization rate, ICU admission, and mechanical ventilation rates from the COVID-19 infection. Overall, our study has shown that receiving the COVID-19 vaccine is beneficial and effective in mitigating the spread of infection and providing better clinical outcomes. We recommend and urge all stakeholders involved in COVID-19 infection prevention, management, and control to strengthen the advocacy and education of the people. All hands should be on deck to ensure that the misconceptions and erroneous beliefs about the effectiveness of the COVID-19 vaccine be corrected based on the scientific evidence from our work and other ongoing research. This will go a long way in reducing the morbidities from COVID-19 and other associated impacts of the COVID-19 pandemic.
